# Late Ileocolic Anastomotic Stricture Due to Subserosal Lipoma: A Case Report

**DOI:** 10.7759/cureus.93976

**Published:** 2025-10-06

**Authors:** Yoichi Miyaoka, Shingo Shimada, Kazuhiro Ogasawara, Akinobu Taketomi

**Affiliations:** 1 General Surgery, Abashiri-Kosei General Hospital, Abashiri, JPN; 2 Surgery, Otaru General Hospital, Otaru, JPN; 3 Surgery, Kushiro Rosai Hospital, Kushiro, JPN; 4 Gastroenterological Surgery, Hokkaido University Graduate School of Medicine, Sapporo, JPN

**Keywords:** anastomotic stricture, ebd, endoscopic balloon dilation, extramural compression, ileocolic anastomosis, non-traversable stricture, subserosal lipoma, ‘surgical resection’

## Abstract

Anastomotic strictures after ileocolic surgery are most often related to technical, ischemic, or inflammatory factors, with tumor recurrence also in the differential; a subserosal lipoma arising at the anastomosis is rare. We report a man in his 60s, 10 years after an ileocecal resection for appendicitis with abscess, who presented with right-sided abdominal pain and repeated vomiting. Contrast-enhanced CT demonstrated small-bowel wall hyperenhancement with fluid retention and a tight narrowing at the ileocolic anastomosis. After stabilization, colonoscopy showed edematous, ulcerated mucosa and a non-traversable stricture; biopsies revealed only inflammatory changes. Fluoroscopic balloon dilation was attempted, but guidewire cannulation failed, and conservative management was judged unlikely to succeed; therefore, the anastomotic segment was resected. Gross examination revealed compression of the bowel wall by subserosal adipose tissue with blunting of mucosal folds. Histology showed nodular proliferation of mature adipocytes in the subserosa without atypia or lipoblasts, consistent with a benign subserosal lipoma. Recovery was uneventful, and the patient was discharged on postoperative day 15. This case highlights subserosal lipoma at an ileocolic anastomosis as an uncommon cause; when endoscopic passage is impossible and imaging suggests an extraluminal fatty component, early surgery should be considered.

## Introduction

Postoperative anastomotic stricture is a clinically relevant problem after ileocolic and colorectal surgery, with reported incidence varying widely (roughly 2-30%) according to definitions, anatomic site, and follow-up duration [1-3]. Major etiologies include technical factors (e.g., anastomotic diameter or tension), ischemia or leak-related scarring, and inflammatory conditions; in oncologic settings, recurrent tumor must also be excluded [1-3]. Strictures may present months to years after surgery and can lead to abdominal pain, vomiting, and obstructive symptoms that complicate endoscopic evaluation and delay definitive management.

Colonic lipomas are uncommon benign adipocytic tumors (estimated prevalence ~0.2-4.4%), the great majority (~90%) arising from the submucosa and projecting intraluminally; subserosal lesions constitute a minority [4,5]. Most symptomatic cases in the literature involve intraluminal bulk effects-bleeding, intermittent obstruction, or intussusception, where endoscopic visualization is often possible [4-6]. By contrast, extramural (subserosal) lipomas may present primarily through external compression, are more difficult to appreciate endoscopically, and are only sparsely represented in reviews. Within this context, a subserosal lipoma developing at a prior anastomosis and manifesting as a fixed, non-traversable anastomotic stricture appears exceedingly rare. This case highlights a distinct diagnostic challenge, as the extramural fatty component can mimic fibrotic or inflammatory stricture without mucosal abnormality.

We report a late ileocolic anastomotic stricture caused by a subserosal lipoma at a previous anastomosis, detailing the imaging-pathologic correlation and discussing practical implications for diagnosis and management. Unlike the more common intraluminal submucosal lipomas, a subserosal lipoma at a surgical anastomosis poses a diagnostic challenge because it produces extramural compression that may mimic fibrotic or inflammatory stricture without mucosal abnormality.

## Case presentation

**Figure 1:**
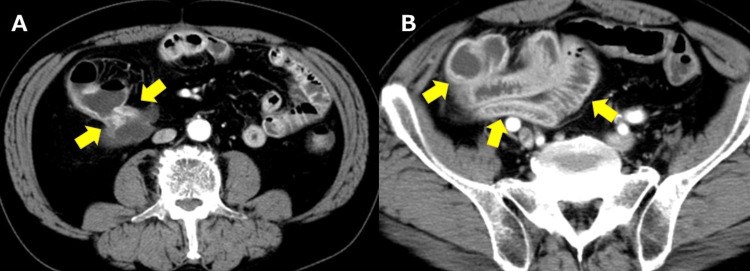
Contrast-enhanced abdominal CT. (A, B) Axial images show a tight ileocolic anastomotic stricture with upstream small-bowel dilatation and mural hyperenhancement; low-attenuation tissue contiguous with the anastomosis (arrows) suggests an extramural fatty component.

A man in his 60s with a history of ileocecal resection for appendicitis with abscess 10 years earlier presented with right-sided abdominal pain and repeated vomiting. On arrival, he was afebrile with localized right-flank tenderness and no peritoneal signs; laboratory tests showed a mild inflammatory response without organ dysfunction. Contrast-enhanced CT demonstrated a high-grade ileocolic anastomotic stricture with a fat-attenuating extramural component contiguous with the anastomosis (Figure 1A) and upstream small-bowel dilatation (Figure 1B).

The extramural component showed homogeneous fat attenuation with Hounsfield Unit values around -90 to -100 HU, consistent with mature adipose tissue. The patient had no prior postoperative imaging between the initial ileocecal resection and the current presentation. Upon admission, conservative therapy consisting of bowel rest and intravenous fluids was attempted for three days without improvement. The original anastomosis had been constructed in a side-to-side stapled fashion.

**Figure 2:**
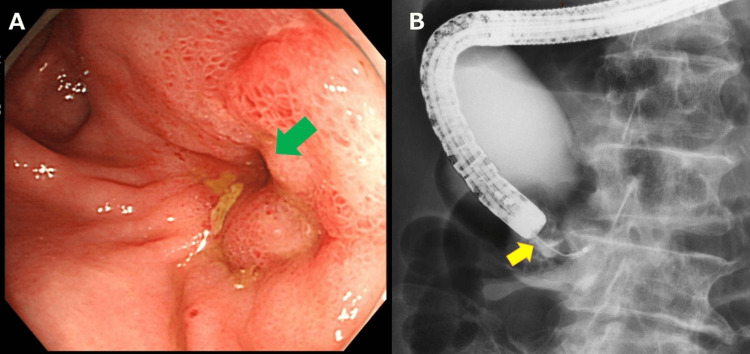
Lower endoscopy and fluoroscopic contrast study. (A) Colonoscopy shows edematous, ulcerated mucosa and a non-traversable anastomosis (green arrow). (B) During attempted balloon dilation under fluoroscopy, guidewire could not be advanced across the stricture and the procedure was aborted; contrast pools proximally (yellow arrow).

After stabilization, colonoscopy revealed edematous, ulcerated mucosa at a non-traversable anastomotic stricture (Figure 2A); mucosal biopsies showed inflammatory changes only. Under fluoroscopic guidance, balloon dilation was attempted, but guidewire cannulation across the stricture failed, and the procedure was aborted (Figure 2B).

Given the non-traversable nature of the lesion and the low likelihood of success with conservative/endoscopic measures, segmental resection, including the anastomosis, was performed.

**Figure 3:**
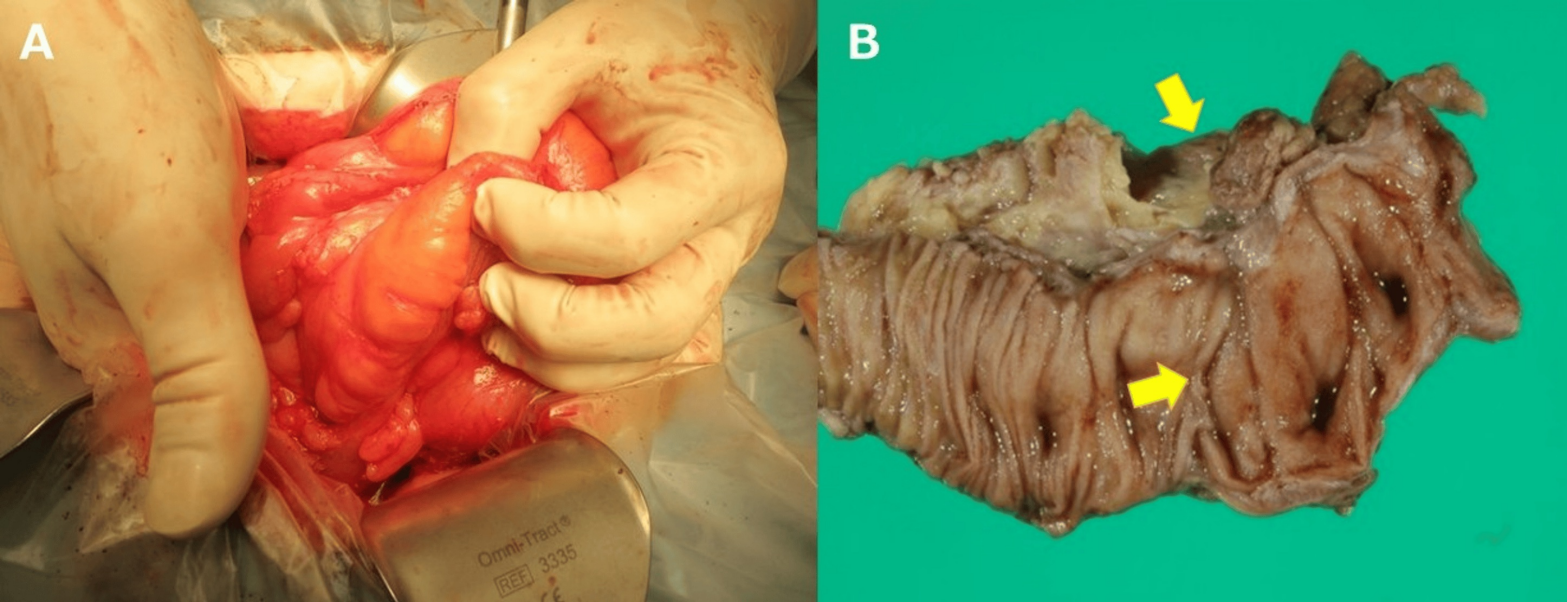
Operative and gross findings. (A) Intraoperative view of the circumferentially thickened and constrictive anastomotic segment. (B) Resected specimen shows extramural (subserosal) adipose tissue compressing the wall with blunted mucosal folds (arrows).

Intraoperatively, the anastomotic segment was circumferentially thickened and constrictive (Figure 3A). The resected specimen showed extramural (subserosal) adipose tissue compressing the bowel wall with blunted mucosal folds (Figure 3B).

**Figure 4:**
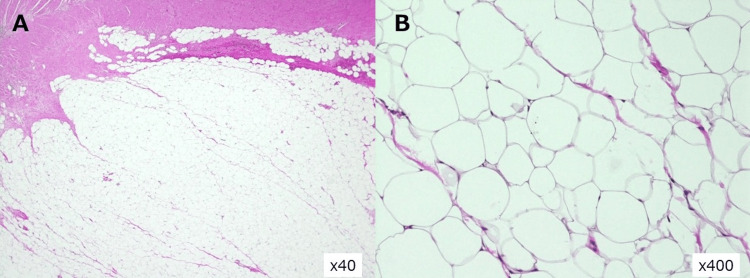
Histopathology (H&E). (A) ×40: subserosal lipomatous proliferation compressing the muscularis-mucosal layers. (B) ×400: mature adipocytes without atypia or lipoblasts, consistent with a benign subserosal lipoma.

Histopathology (H&E) demonstrated nodular proliferation of mature adipocytes in the subserosa without atypia or lipoblasts, consistent with a benign subserosal lipoma (Figure 4).

Oral intake resumed on postoperative day (POD) 1, diet advanced on POD 5, and the patient was discharged in good condition on POD 15.

## Discussion

Late ileocolic anastomotic stricture is most often attributable to technical factors, ischemia, or leak-related scarring, inflammatory disease, or prior radiation; in oncologic settings, recurrent tumor must also be excluded [1-3]. In our patient, 10 years after ileocecal resection, the triad of a non-traversable anastomotic narrowing, biopsy-negative mucosa, and fat attenuation contiguous with the anastomosis on CT favored an extramural rather than intrinsic scar etiology. Resection confirmed a subserosal lipoma compressing the bowel without atypia. To the best of our knowledge, no previous reports have described an extramural (subserosal) lipoma developing at a gastrointestinal anastomotic site, making this case a unique presentation.

Table 1 summarizes the present case alongside representative case reports and reviews, emphasizing the layer of origin, anatomic site, endoscopic traversability, and management.

**Table 1:** Summary of related literature (present case + case reports/reviews). The table highlights layer of origin, anatomic site, endoscopic traversability, and management. Most lesions are submucosal and intraluminal; the present case uniquely involves a subserosal (extramural) lipoma at a prior ileocolic anastomosis, causing a fixed, non-traversable stricture requiring resection. CR, case report; GW, guidewire.

Authors	Country	Type	Year	Site and Layer	Presentation	Endoscopic Traversal	Management	Relevance to Present Case
Present case	Japan	CR	2025	Ileocolic anastomosis; subserosal	Fixed, non-traversable anastomotic stricture with pain/vomiting	Not traversable; biopsy inflammatory only; GW failed	Segmental resection incl. anastomosis; benign subserosal lipoma	Index case showing extramural (subserosal) lipoma at an anastomosis causing fixed stricture
Bronswijk et al. [4]	Belgium	Systematic review	2020	Colon; predominantly submucosal, intraluminal	Obstruction/intussusception (bleeding less common)	Often traversable; variable	Endoscopic resection; surgery for broad-based/complicated lesions	Size/morphology-based selection framework; literature mainly intraluminal
Crocetti et al. [5]	Italy	Systematic Review	2014	Colon; symptomatic lipomas	Larger size → more obstruction/intussusception	Variable (case-dependent)	Surgery favored when large/broad-based/complicated	Supports surgery in obstructive settings
Jiang et al. [6]	China	CR	2007	Colon; submucosal giant lipoma	Obstruction from intraluminal mass	Usually visible/traversable endoscopically	Segmental resection	Intraluminal (submucosal) mechanism vs. our extramural (subserosal)
Mouaqit et al. [7]	Morocco	CR	2013	submucosal; intraluminal	Obstruction due to intussusception	Not reported	Surgical resection	Comparator - intraluminal/intussuscepting lipoma vs. our extramural
Fiordaliso et al. [8]	Italy	Narrative review	2024	Colonic lipoma overview	Patterns of obstruction/intussusception	Context-dependent	Algorithms for endoscopic vs. surgical strategies	Contemporary framework; anastomosis-specific cases rare

Most symptomatic colonic lipomas in prior reports are submucosal and intraluminal, often visible endoscopically and sometimes traversable, with obstruction driven by bulk or intussusception-typified by the case reports of Jiang et al. and Mouaqit et al. [6,7]. By contrast, our case derives from the subserosa (extramural) at a prior anastomosis, producing a fixed, non-traversable stenosis - an anatomic context that appears rarely documented. Reviews by Bronswijk and Crocetti et al. emphasize size/morphology-based selection between endoscopic and surgical therapy [4,5], while the contemporary narrative review by Fiordaliso et al. outlines patterns of obstruction/intussusception and treatment algorithms but likewise contains few anastomosis-specific examples [8]. Read alongside these sources, the present case broadens the differential for late anastomotic narrowing to include extramural (subserosal) lipoma.

From a diagnostic standpoint, CT characteristics of lipoma-homogeneous fat attenuation with minimal enhancement help distinguish it from other subepithelial lesions (SELs); when the fatty signal is contiguous with the anastomosis, it argues against a simple inflammatory scar and toward a lipomatous extramural process [4,7,9-11]. When clinical status allows, MRI can serve as a problem-solving adjunct: lipomas typically show high T1/T2 signal with marked signal loss on fat suppression and on opposed-phase imaging, aiding confirmation of fat, assessment of the layer of origin (submucosal vs. subserosal), and evaluation for any non-fatty enhancing components suggestive of alternative pathology (e.g., well-differentiated liposarcoma) [12,13]. In our patient, an MRI was not obtained because of acute obstruction, and the CT triad (fat contiguous with the anastomosis, non-traversable stricture, negative biopsies) already supported an extramural lipomatous etiology and prompted early surgery. Routine mucosal biopsies have low yield for deeper or extramural pathology, as guidance on SELs underscores [10,11].

Therapeutically, endoscopic balloon dilation (EBD) achieves high immediate success for benign colorectal or ileocolic strictures, but outcomes are poor when the stricture is non-traversable or caused by extrinsic compression [1,2,8,14-16]. In our patient, failed guidewire passage during attempted dilation predicted low endoscopic success and justified early surgery, which provided both definitive diagnosis and curative relief. Consistently, the systematic review by Crocetti et al. supports surgery for large/broad-based or complicated lipomas, and both Jiang and Mouaqit ultimately required segmental resection for obstructive disease despite intraluminal (submucosal) origin [5-7].

Although the concept of post-traumatic adipocytic proliferation has been described in soft-tissue literature [17], its application to gastrointestinal anastomoses remains speculative and should be interpreted cautiously.

The anastomotic interface is characterized by chronic scarring and low-grade mechanical irritation around sutures or staples, conditions that may provide a permissive niche for adipocytic proliferation in the subserosa. While still speculative in the gastrointestinal tract, this framework plausibly explains the emergence of an extramural lipoma at a prior anastomosis and the resulting fixed, non-traversable stenosis. Given the potential for extramural pathology to mimic fibrotic or inflammatory strictures, multidisciplinary evaluation - integrating radiologic pattern recognition (fat-attenuating components on CT/MRI), endoscopic assessment of traversability, and surgical judgment - can expedite accurate diagnosis and definitive treatment.

Putting this together, a practical heuristic for late anastomotic strictures is (i) scrutinize CT for a fat-attenuating component contiguous with the anastomosis; (ii) if the stricture is non-traversable and guidewire passage fails, prioritize operative management over repeated EBD; and (iii) in oncologic contexts, continue to exclude tumor recurrence in parallel [1-6,10,11].

This approach aligns with the aggregate signal from the literature while acknowledging that our case is an extramural, anastomosis-specific outlier relative to the predominantly intraluminal reports.

Limitations include the single-case nature of this report, the absence of (endoscopic ultrasound) EUS due to anatomic constraints at the ileocolic anastomosis, qualitative rather than prospective quantitative HU assessment, and the inability to pinpoint the exact site of origin within the adjacent bowel on the specimen. Nevertheless, the clinical course, imaging, and histology are coherent and mutually reinforcing.

## Conclusions

This case suggests that when CT demonstrates a fat-attenuating lesion contiguous with a non-traversable anastomosis and biopsies are negative, early surgical resection may be preferable to repeated dilation. Multidisciplinary evaluation - combining radiologic pattern recognition and endoscopic assessment of traversability - can expedite accurate diagnosis and treatment. 
